# Effects of extra training with active cycle of breathing technique relative to whole-body breathing exercise in older adults with stable COPD

**DOI:** 10.1371/journal.pone.0349597

**Published:** 2026-06-08

**Authors:** Huiping Lai, Lanzhen Chen, Roger W. Chan, Qiongrong Pan, Qing Chen, Ying Chen, Zhenyu Xie

**Affiliations:** 1 School of Nursing, Xiamen Medical College, Xiamen, Fujian, China; 2 Geriatric Care Research Center, Xiamen Medical College, Xiamen, Fujian, China; 3 Department of Nursing, Second Affiliated Hospital, Xiamen Medical College, Xiamen, Fujian, China; 4 School of Basic Medicine, Xiamen Medical College, Xiamen, Fujian, China; 5 Institute of Respiratory Diseases, Xiamen Medical College, Xiamen, Fujian, China; 6 Department of Respiratory Medicine, Second Affiliated Hospital, Xiamen Medical College, Xiamen, China; University of Insubria: Universita degli Studi dell’Insubria, ITALY

## Abstract

**Objective:**

To examine the effects of extra training with active cycle of breathing technique (ACBT) as compared to whole-body breathing exercise (WBBE) in older adults with stable chronic obstructive pulmonary disease (COPD).

**Methods:**

Fifty-one participants with stable COPD were randomly assigned into experimental group undergoing extra training of ACBT plus WBBE (*n* = 25), and control group undergoing WBBE only (*n* = 26), 30 minutes daily for 3 months. Lung function including forced expiratory volume in one second (FEV1), FEV1 in percentage of predicted value (FEV1%) and FEV1/forced vital capacity (FVC) ratio; exercise capacity (six-minute walk test distance, 6MWD); dyspnea symptoms (modified Medical Research Council, mMRC dyspnea scale score and Dyspnoea-12-Chinese/D-12-C scale score); and quality of life in terms of COPD Assessment Test (CAT) score before and after intervention were evaluated.

**Results:**

Significantly higher FEV1 (1.82L versus 1.76L, *p* < 0.05) was found for the experimental group, with no significant differences in FEV1% and FEV1/FVC ratio. Significantly larger 6MWD (359.72m versus 340.18m, *p* < 0.001), significantly lower D-12-C scores (11.47 versus 13.55, *p* < 0.001), and significantly lower CAT scores (2.95 versus 3.98, *p* < 0.001) were observed in the experimental group relative to the control group.

**Conclusions:**

Our findings suggested that ACBT in conjunction with WBBE could be effective complementary approaches for improving lung function and exercise capacity, reducing dyspnea symptoms, and enhancing quality of life in older adults with stable COPD. Future studies may explore how ACBT and WBBE could be optimized in training frequency, intensity, and duration specifically for older adults.

## Introduction

Pulmonary rehabilitation is standard treatment for patients with chronic obstructive pulmonary disease (COPD) according to the Global Initiative for Chronic Obstructive Lung Disease (GOLD), an international organization dedicated to improving the diagnosis, management, and prevention of COPD based on the latest scientific evidence, in particular for those with respiratory distress symptoms as routine non-pharmacological intervention [1]. Pulmonary rehabilitation is effective for COPD patients with dyspnea symptoms in the stable phase, as well as for those with debilitating acute exacerbations that severely affect activities of daily living [2,3]. Dyspnea and excessive airway mucus are actively managed to mitigate respiratory symptoms with therapy tailored to individual needs, including targeting triggers that would lead to acute exacerbations [4]. A key component of pulmonary rehabilitation is exercise training, a cornerstone for improvements in exercise capacity, endurance and tolerance [3,5]. Exercise training typically involves general physical conditioning exercises, cardiovascular fitness and aerobic exercises, as well as specific respiratory/breathing exercises [3,5]. This study focused on evaluating the approach of active cycle of breathing technique (ACBT), in conjunction with whole-body breathing exercise (WBBE) [6]. ACBT was originally developed as a forced breathing exercise to facilitate airway clearance for individuals with abnormal/excessive airway secretions, including cystic fibrosis, non-cystic fibrosis bronchiectasis, and COPD [7,8]. ACBT is composed of three main components or phases, including cycles of breathing control, thoracic expansion exercises, and forced expiratory technique (FET), with an emphasis on relaxed breathing interspersed in between exercises [8–11]. An ACBT cycle typically consists of breathing control, 3–4 thoracic expansion exercises, breathing control, and FET. An integral part of ACBT is the FET, consisting of forced expirations or “huffs” together with thoracic expansion exercises and periods of breathing control (relaxed breathing) [9–11]. On the other hand, WBBE involves exercises of non-respiratory muscles (muscles of the whole body, hence its name) in conjunction with respiratory muscle training, typically including the following: relaxation exercises; abdominal/diaphragmatic breathing exercises; arm stretching, extension and flexion exercises; chest-hugging exercises; and knee-bending exercises; with all of them performed while emphasizing coordination with inhalation and exhalation [6].

While ACBT can effectively clear abnormal and excessive airway secretions, its effects are focused on breathing and do not necessarily reach other parts of the body [9]. As an approach to address non-respiratory muscle training, WBBE could be an effective complement to ACBT as it targets both respiratory and non-respiratory (limb) muscles [5,6]. The objective of this study was to evaluate the effects of ACBT in conjunction with WBBE on key patient-oriented outcomes in older adults with stable COPD. Specifically, our focus was to evaluate the impact of extra training of ACBT by comparing the effects of ACBT plus WBBE versus WBBE alone. It was hypothesized that the combined training effects relative to WBBE alone could be superior as both respiratory and non-respiratory muscles were involved in the training.

## Materials and methods

### Participants

Fifty-eight community-dwelling older adults were initially enrolled in the study. Inclusion criteria included: (1) formal diagnosis of COPD based on the Chinese Thoracic Society’s Guidelines [6], with post-bronchodilator forced expiratory volume in one second (FEV1)/forced vital capacity (FVC) ratio<70% plus exclusion of other respiratory disease diagnoses [1]; (2) being in the stable phase of COPD [6]; (3) age at or above 50 years old; (4) independent mobility (walking independently for 45 m) with or without assistive devices/walking aids; (5) being prescribed similar dosages of pulmonary medications including bronchodilators; (6) stable medical conditions, ability to follow clinician instructions in completing rehabilitation exercises. Exclusion criteria included: (1) being in the acute/exacerbation phase of COPD [6]; (2) history of severe neurological disorders, physical, cognitive or intellectual disabilities, inability to comply with instructions for completing rehabilitation tasks; (3) previous participation in pulmonary rehabilitation or other behavioral interventions. All 58 participants were native Chinese, with 7 dropping out after one month because of poor adherence (scheduling conflicts; 4 from the experimental group and 3 from the control group). The remaining 51 participants completed the intervention, with gender and age distributions (means ± SD in years) of 11 females (63.95 ± 5.40) and 14 males (69.86 ± 6.14) in the experimental group, and 11 females (66.36 ± 7.75) and 15 males (70.53 ± 5.07) in the control group. Twenty-three of 25 participants in the experimental group and 24 of 26 participants in the control group were above 60. Independent-samples *t t*ests were conducted to evaluate if there were any differences in age across the two participant groups, and also to evaluate if there were any group differences in severity of COPD, i.e., GOLD stages 1–4 classification based on post-bronchodilator FEV1% (percentage of predicted value) [1].

### Experimental procedure

**Fig 1:**
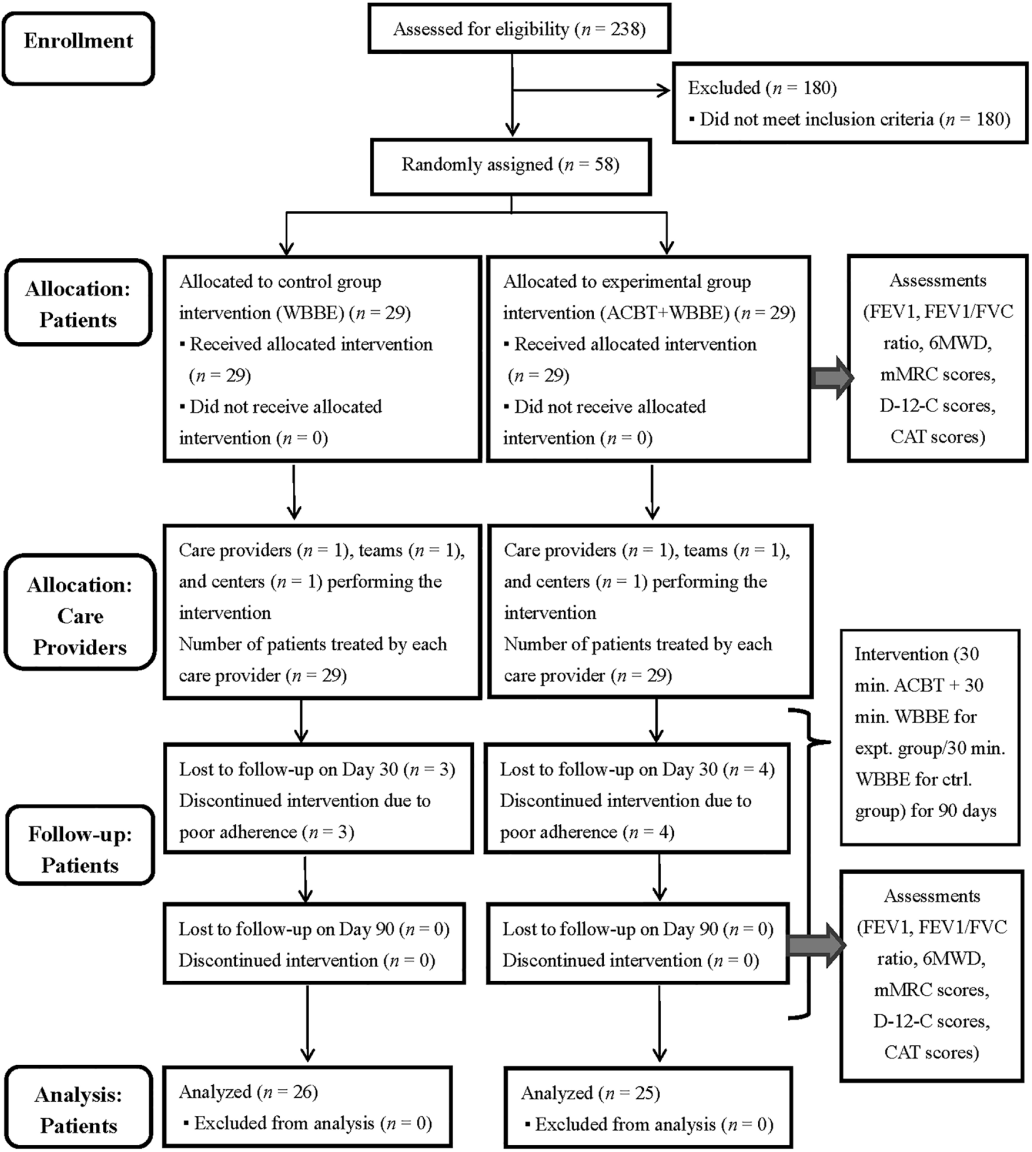
Experimental procedure: passage of participants through the study based on updated Consolidated Standards of Reporting Trials (CONSORT) guidelines [17].

The experimental protocol was approved by the Institutional Review Board (Ethics Review Committee) of Second Affiliated Hospital, Xiamen Medical College (approval number 2023094). Written informed consents were obtained from all participants. The recruitment period started on 2023/11/12 and ended on 2023/12/7. Participants underwent pre-intervention assessments of lung function (FEV1, FEV1% and FEV1/FVC ratio) [12], exercise capacity (six-minute walk test distance, 6MWD) [13], dyspnea symptoms (modified Medical Research Council, mMRC dyspnea scale score [14] and Dyspnoea-12 scale score [15]), and quality of life (QOL) (COPD Assessment Test, CAT score) [16]. Participants were randomly assigned into (1) experimental group (*n* = 25), who underwent ACBT plus WBBE intervention (one 30-minute session of ACBT training and one 30-minute session of WBBE training daily, with the sessions separated by at least 30 minutes of rest, for approximately 90 days); and (2) control group (*n* = 26), who underwent WBBE intervention only (one 30-minute session of WBBE training daily, for approximately 90 days). Randomization was performed with a computer-generated random number sequence created by an investigator unaware of the identity of the participants being assigned, to randomly assign participants to either of the two groups using a maximum tolerated imbalance value of 3 with the asymptotic maximal procedure. Allocation concealment was ensured with the random number sequence concealed in sequentially numbered, opaque sealed envelopes. Immediately following intervention, post-intervention assessments of outcome measures were conducted. Participants were blinded to their group assignment, but the nature of the intervention made true blinding of the participants and the trainers delivering the intervention difficult, which could lead to a risk of performance bias. Nevertheless, outcome assessors (for lung function, 6MWD, and BODE measures) were blinded to participants’ group allocation, which helped ensure a low risk of detection bias for the objectively determined outcome measures. Fig 1 shows the procedure following updated CONSORT reporting guidelines [17].

### Assessments

#### (1) Lung function.

FEV1 (in L), FEV1% and FEV1/FVC ratio were measured by a pulmonary test system (Vitalograph Model 6000 Alpha, Ireland) calibrated according to ISO26782 recommendations. Following standard procedure of the American Thoracic Society and European Respiratory Society [18], and standardized adult spirometry in China [19], each participant was comfortably seated and performed forced expiratory maneuvers consisting of maximal inspiration, maximal forced expiration, and maximal inspiration after maximal forced expiration. FVC (in L) was determined as the maximum amount of air that was exhaled (volume of air exhaled until plateau in the FVC curve), FEV1 (in L) was the volume of air exhaled in the first second in the FVC curve, FEV1% was FEV1 in percent of predicted value, and FEV1/FVC ratio was also in percentages. There were 3 trials for obtaining all data, with 1–2 minutes rest periods in between trials to ensure participant recovery. Repeatability was achieved with differences between the largest and next largest FEV1 (and FVC) values ≤ 0.15L, and trials with the highest values were taken [18]. All measurements were conducted by a trained technician with more than 5 years of clinical experience in lung function assessment, carried out in the afternoon to minimize variability [19].

#### (2) 6MWD.

Functional exercise capacity was determined by six-minute walk test (6MWT), following official guidelines [13]. 6MWT is a widely used, safe, and reliable field-based walking test for evaluating cardiopulmonary function, with 6MWT distance (6MWD) reflecting the level of exercise capacity and tolerance [20]. Briefly, each participant walked according to line markings on an open and flat 30-m long corridor, covering as much distance as possible in 6 minutes, with standard instructions and feedback of encouragement given. The test was repeated twice as recommended [20].

#### (3) mMRC dyspnea scale scores.

The mMRC dyspnea scale is a well validated, straightforward psychophysical scale to evaluate one’s perceived symptoms and severity of dyspnea during activities of daily living, or the degree of functional disability due to respiratory distress [14,21]. Patients would grade their perceived level of discomfort/degree of respiratory distress with a 5-point Likert scale (0–4), with higher grades indicating more severe distress affecting activities of daily living. It is widely used for evaluation of dyspnea in COPD patients, being well correlated with 6MWD, FEV1, and prognosis [22]. The Chinese version of the scale is widely used for evaluation of dyspnea in Chinese-speaking populations [23].

#### (4) BODE index.

BODE measures for all participants were evaluated, including body-mass index (B), degree of airflow obstruction (O) as determined by FEV1%, mMRC score (D), and exercise capacity (E) as determined by 6MWD. Based on these measures, the BODE index was calculated according to the unique scoring system devised by Celli et al., which was designed to simultaneously quantify pulmonary impairment, perceived dyspnea, and systemic consequences of COPD (BMI and 6WMD) by combining these outcome variables across different domains [12,24]. The BODE index has been widely used in the literature as a well-established prognostic indicator for estimating mortality from respiratory causes in COPD patients [12]. It could predict the risk of death, hospitalizations and exacerbations of COPD better than FEV1, and also indicate the severity of COPD [12,24].

#### (5) Dyspnoea-12-Chinese (D-12-C) scale scores.

The D-12 scale is widely used to evaluate the symptoms and severity of dyspnea [15]. The scale could address the multidimensional nature of respiratory and dyspnea symptoms beyond what is examined with the shorter mMRC scale. With 7 of the 12 items focusing on physical effects and 5 items on affective/psychological effects of dyspnea, and a 4-point Likert scale, D-12 total scores would range from 0–36 points with higher scores indicating more severe dyspnea. D-12 total scores correlate well with mMRC scores, St. George’s Respiratory Questionnaire scores, Hospital Anxiety and Depression Scale scores, and 6MWD [15]. D-12 was cross-culturally adapted into Chinese (abbreviated as D-12-C) and validated with Cronbach’s alpha = 0.88, with D-12-C total scores being moderately correlated with St. George’s Respiratory Questionnaire scores (Spearman’s *rho* = 0.59) and Hospital Anxiety and Depression Scale scores (Spearman’s *rho* = 0.46) [25].

#### (6) CAT scores.

The impact of COPD on QOL was evaluated by CAT scores. Compared with the complicated St. George’s Respiratory Questionnaire limiting its routine clinical use, CAT is a simple, eight-item unidimensional tool for quantifying health status, wellbeing and QOL in individuals with COPD [16]. With items addressing severity of cough, sputum level, chest tightness, degree of breathlessness, limitations in activities at home, confidence in leaving home, sleep quality, and energy level, and a 6-point Likert scale, the impact of COPD is evaluated by total scores of 0−40 with higher scores indicating more severe impact on QOL [16]. The Chinese version of CAT was validated with Cronbach’s alpha = 0.805, being well correlated with FEV1 (Pearson’s *r* = −0.567) [26].

### Intervention (control group)

Participants in the control group underwent WBBE for 30 minutes daily, for approximately 90 days. WBBE was conducted following official guidelines [6]. Training workshops were organized for groups of participants who were recruited at around the same times, with standard instructional videos on specific exercises: (1) warm-up relaxation exercises; (2) abdominal/diaphragmatic breathing exercises, with a 1:3 ratio in inhalation to exhalation duration; (3) arm stretching exercises with inhalation/exhalation; (4) crossed-arm exercises with inhalation/exhalation; (5) arm extension and raising exercises with inhalation, arm flexion and lowering with exhalation; (6) chest-hugging exercises with inhalation/exhalation; and (7) knee-bending exercises with inhalation/exhalation.

### Intervention (experimental group)

In addition to WBBE, participants in the experimental group underwent ACBT for 30 minutes daily, for approximately 90 days. The specific ACBT cycle adopted followed recommendations of Pryor et al. [8], with an emphasis on the necessity for relaxed breathing interspersed with thoracic expansion exercises and FET for older adults [9–11]: (1) relaxed breathing; (2) 1–2 sets of FET (forced expirations/huffing), involving forceful exhalation with glottal opening, followed by effective coughing and sputum excretion; (3) relaxed breathing; (4) 3–4 sets of thoracic expansion exercises; and (5) relaxed breathing, with an emphasis on interspersed relaxed breathing. There were 5 repetitions per series (requiring 5 minutes to complete a series on average), followed by a rest period of at least 1 minute with relaxed breathing, for a total of 5 series, resulting in a total training duration of approximately 30 minutes daily. Participants’ adherence to the training was monitored by regular social media contacts and phone calls regarding their log sheet records of the training activities completed.

### Statistical analysis

Statistical analysis was conducted with SPSS 26.0 (IBM), with alpha = 0.05 (two-tailed). Descriptive statistics were computed for all outcome measures. The assumption of normality of distribution for the data was first verified by the Shapiro-Wilk test, revealing no strong evidence of violation (for example, Shapiro-Wilk *W* statistic for the pre-intervention outcome measures ranged from 0.94 to 0.98, *p* > 0.05). To compare the effects of intervention across groups, analysis of covariance (ANCOVA) was conducted to determine if there were significant between-group differences in post-intervention outcome measures while controlling for (adjusted for) the pre-intervention baseline measures as covariates [27]. For the analysis of categorical (ordinal) variables, i.e., mMRC, D-12-C and CAT scores, they were first transformed from ordinal to continuous variables based on a classical ordinal encoding procedure with 5(0–4) encodings, 37(0–36) encodings, and 41(0–40) encodings for mMRC, D-12-C and CAT scores, respectively [28,29]. Adjusted mean values of outcome measures were generated with ANCOVA to reflect their magnitude post-intervention. To evaluate correlations among outcome measures post-intervention, Pearson’s *r* was calculated among those measures demonstrating significant differences between the participant groups.

## Results

Results of independent-samples *t* tests demonstrated no significant differences in age between the two participant groups (23 of 25 participants in the experimental group and 24 of 26 participants in the control group were above age 60): *t*(20)=0.85 (*p* = 0.4075) for female participants; *t*(27)=0.32 (*p* = 0.7481) for male participants; and also no significant group differences in severity of COPD, i.e., GOLD stages 1–4 classification based on post-bronchodilator FEV1% (percentage of predicted value) [1]: 1.48 ± 0.71 for experimental group versus 1.92 ± 1.09 for control group (*t*(49)=1.71, *p* = 0.0942). Table 1 summarizes BODE measures for the participant groups prior to intervention. Results of independent-samples *t* tests (two-tailed) revealed no significant differences in all BODE measures and in the BODE index between the two groups (*p* > 0.05), suggesting comparable participant groups in nature and severity of COPD pre-intervention [12,24].

**Table 1:** BODE measures in two participant groups before intervention (means ± SD; n = 51), with results of independent-samples t tests across groups.

BODE Measures	Expt Group (*n* = 25)	Ctrl Group (*n* = 26)	*t* (49)	*p*
BMI (kg/m^2^)	24.18 ± 2.60	23.09 ± 3.24	1.30	0.200
FEV1%	75.64 ± 19.11	66.14 ± 17.47	1.85	0.070
mMRC score	1.08 ± 0.76	1.12 ± 1.03	−0.14	0.890
6MWD (m)	310.36 ± 33.33	301.535 ± 30.82	0.98	0.331
BODE index	1.76 ± 1.09	2.27 ± 1.69	−1.28	0.208

Note: BODE = body-mass index (B), degree of airflow obstruction (O), dyspnea symptoms (D), and exercise capacity (E); BMI = body-mass index; FEV1% = forced expiratory volume in one second (in percentage of predicted value); mMRC = modified Medical Research Council dyspnea scale; 6MWD = six-minute walk test distance.

### Changes in outcome measures

**Fig 2:**
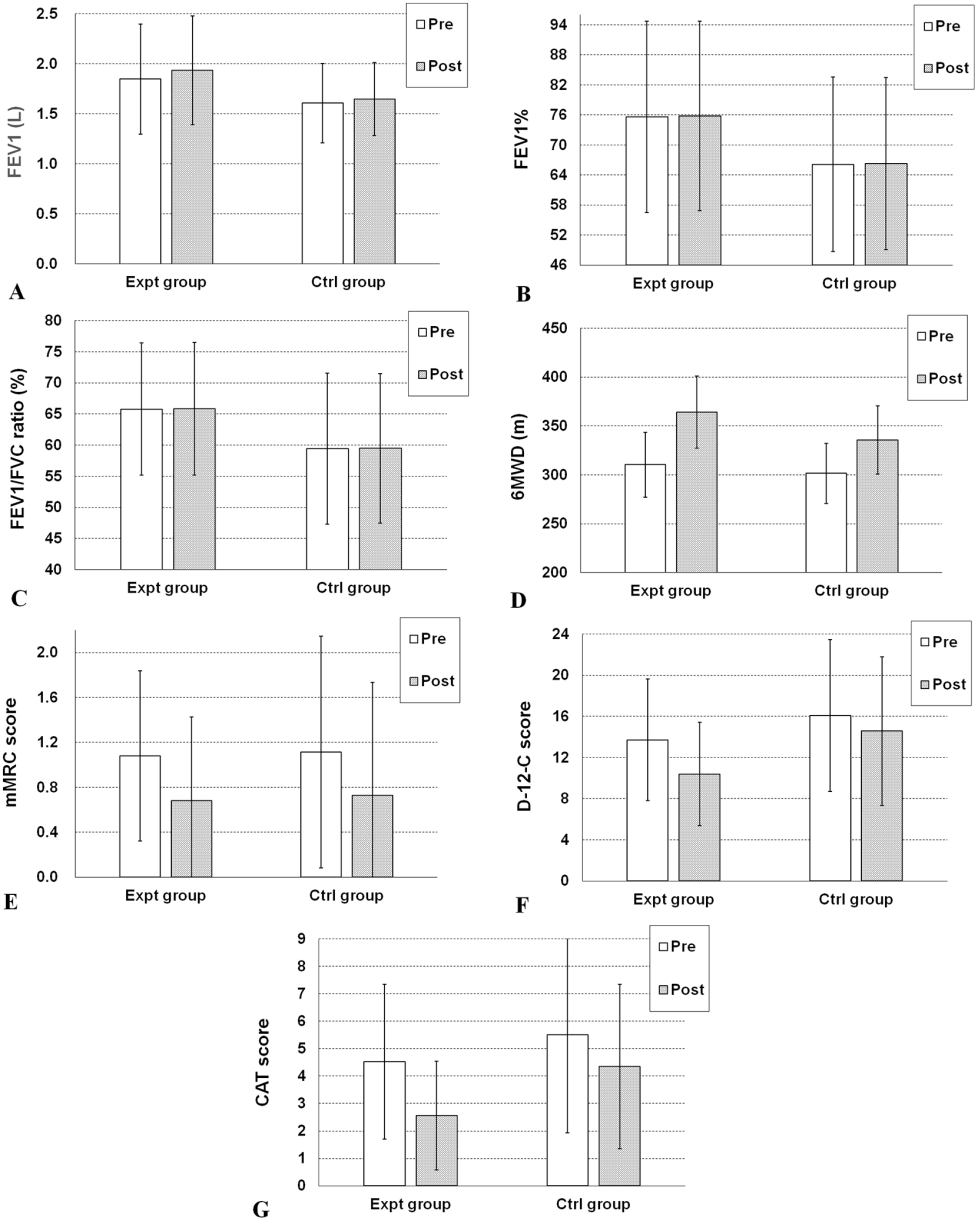
Effects of intervention on changes in outcome measures (means ± SD) before (Pre) and after (Post) intervention in two participant groups (*n* = 25 for experimental group; *n* = 26 for control group): (A) FEV1 (forced expiratory volume in one second) in L (*p* < 0.05); (B) FEV1% (FEV1 in percentage of predicted value) (*p* > 0.05); (C) FEV1/FVC (forced vital capacity) ratio (*p* > 0.05); (D) 6MWD (six-minute walk test distance) (*p* < 0.001); (E) mMRC (modified Medical Research Council dyspnea scale) score (*p* > 0.05); (F) D-12-C (Dyspnoea-12-Chinese scale) score (*p* < 0.001); (G) CAT (COPD Assessment Test) score (*p* < 0.001).

Table 2 shows changes in outcome measures before and after intervention in the participant groups, including FEV1, FEV1%, FEV1/FVC ratio, 6MWD, mMRC scores, D-12-C scores, and CAT scores. ANCOVA was conducted to evaluate effects of the intervention across groups, examining between-group differences with the pre-intervention baselines as covariates. Results indicated significant between-group differences in FEV1 (*p* < 0.05), with a significantly higher FEV1 in the experimental group (adjusted mean = 1.82L versus 1.76L) following intervention; in 6MWD (*p* < 0.001), with a significantly larger walking distance in the experimental group (adjusted mean = 359.72m versus 340.18m); in D-12-C scale scores (*p* < 0.001), with significantly lower scores in the experimental group (adjusted mean = 11.47 versus 13.55); and in CAT scores (*p* < 0.001), with significantly lower scores in the experimental group (adjusted mean = 2.95 versus 3.98) (Table 2). Fig 2 shows the effects of intervention on changes in outcome measures for both participant groups.

**Table 2:** Changes in outcome measures before (Pre) and after (Post) intervention in two participant groups (means ± SD; *n* = 51), with analysis of covariance (ANCOVA) testing for differences across the two groups.

Outcome Measures	Expt Group (*n* = 25)		Ctrl Group (*n* = 26)		Results of ANCOVA^a^	
	Pre	Post (Adjusted Means [95% CIs]) ^a^	Pre	Post (Adjusted Means [95% CIs]) ^a^	*F*(1,48) [*η*_p_^2^]	*p*
FEV1 (L)	1.85 ± 0.55	1.94 ± 0.54 (1.82[1.78, 1.87])	1.61 ± 0.40	1.65 ± 0.37 (1.76[1.72, 1.80])	4.13 [0.08]	0.048 (Expt > Ctrl)
FEV1%	75.64 ± 19.11	75.75 ± 18.92 (75.73[75.60, 75.86])	66.14 ± 17.47	66.29 ± 17.17 (66.36[66.23, 66.49])	0.48 [0.01]	n.s.
FEV1/ FVC ratio (%)	65.79 ± 10.64	65.83 ± 10.65 (62.59[62.50, 62.68])	59.41 ± 12.12	59.47 ± 12.03 (62.58[62.50, 62.67])	0.02 [0.00]	n.s.
6MWD (m)	310.36 ± 33.33	364.30 ± 36.98 (359.72[353.64, 365.79])	301.54 ± 30.82	335.77 ± 34.82 (340.18[334.22, 346.13])	21.12 [0.31]	<0.001 (Expt > Ctrl)
mMRC score	1.08 ± 0.76	0.68 ± 0.75 (0.70[0.50, 0.89])	1.12 ± 1.03	0.73 ± 1.00 (0.72[0.53, 0.91])	0.03 [0.00]	n.s.
D-12-C score	13.72 ± 5.90	10.40 ± 5.03 (11.47[10.73, 12.22])	16.08 ± 7.37	14.58 ± 7.22 (13.55[12.82, 14.28])	15.69 [0.25]	<0.001 (Expt < Ctrl)
CAT score	4.52 ± 2.82	2.56 ± 1.98 (2.95[2.71, 3.18])	5.50 ± 3.57	4.35 ± 2.99 (3.98[3.75, 4.20])	40.53 [0.46]	<0.001 (Expt < Ctrl)

*Note:* The designation “Expt> Ctrl” (or “Expt <Ctrl”) indicates that the adjusted mean values of the post-intervention data for the experimental group were significantly higher (or lower) than those for the control group. CIs = confidence intervals; FEV1 = forced expiratory volume in one second; FEV1% = FEV1 in percentage of predicted value; FVC = forced vital capacity; 6MWD = six-minute walk test distance; mMRC = modified Medical Research Council dyspnea scale; D-12-C = Dyspnoea-12-Chinese scale; CAT = COPD Assessment Test; n.s. = nonsignificant.

^a^ANCOVA was conducted to determine if there was a significant difference between the two groups on the post-intervention data while controlling for (adjusted for) the pre-intervention baseline data as covariates, with adjusted mean values of the post-intervention data generated by ANCOVA while taking into account the different pre-intervention data (different baselines) of the two participant groups.

### Correlations among measures

Results of Pearson’s correlation analysis showed that among measures demonstrating significant group differences, there were positive, moderate correlations between FEV1 and 6MWD; between D-12-C scores and CAT scores; and negative, moderate correlations between FEV1 and D-12-C scores as well as CAT scores; between 6MWD and D-12-C scores as well as CAT scores (Table 3).

**Table 3:** Correlations among the outcome measures FEV1, 6MWD, D-12-C scores, and CAT scores (post-intervention data; Pearson’s *r*) (*n* = 51).

Measures	FEV1	6MWD	D-12-C score	CAT score
FEV1	---			
6MWD	0.507***	---		
D-12-C score	−0.400**	−0.674***	---	
CAT score	−0.374*	−0.398**	0.467**	---

*** *p* < 0.001 (two-tailed); ** *p* < 0.005 (two-tailed); * *p* < 0.01 (two-tailed).

## Discussion

The objective of this study was to examine the effects of ACBT combined with WBBE in older adults with stable COPD, focusing on older adults since they make up a significant portion of all patients with stable COPD, especially in China [30]. This study focused specifically on the combined training, hypothesizing that the complementary effects of extra training of ACBT plus WBBE relative to WBBE alone could be more effective for training both respiratory and non-respiratory muscles, leading to better improvements in outcome measures. Our findings of mostly larger improvements in outcome measures with the extra training of ACBT in the experimental group were generally consistent with the positive effects of ACBT in the literature [9–11].

### Effect of ACBT in combination with WBBE

Results showed that following intervention of approximately 90 days, there were significant improvements in the experimental group in multiple measures. Firstly, a significantly higher FEV1 (in L) indicated improved lung function, despite of no significant difference in FEV1%. However, although this difference in FEV1 between the two groups was statistically significant, the magnitude (0.06L on average) was only modest in clinical terms. We speculate that given the lack of significant group difference in FEV1/FVC ratio, the difference in FEV1 could reflect the primary effects of ACBT being on respiratory muscle performance and ventilatory efficiency (perhaps through FET training in particular), rather than structural airway changes and changes in airflow obstruction. A recent study demonstrated that higher FEV1 could be associated with lower mortality even for FEV1 values in the normal range, suggesting that higher FEV1 is a “biomarker of better health” [31]. Nonetheless, this was only speculative and any such effects of ACBT on respiratory muscles should be examined further in future studies designed specifically to address the potential underlying mechanisms. On exercise capacity, with 6MWD being a well-established metric responsive to intervention, significantly larger 6MWD suggested improvements in exercise capacity that would be well correlated with improvements in other domains (lung function and QOL) [20]. Importantly, for both participant groups the observed changes in 6MWD with intervention exceeded the minimum clinically important difference of 35 meters for COPD patients, where 6MWD increased by 49.4m for the experimental group and by 38.6m for the control group, on average (both beyond a 10% change in the baseline 6MWD) (Table 2; Fig 2D) [32]. This finding together with the observed between-group difference of 19.5m post-intervention not reaching the minimum clinically important difference suggested that both groups achieved comparable, clinically important improvements in exercise capacity [32]. On dyspnea symptoms during activities of daily living, given the fact that mMRC scores and D-12-C scores are complementary in nature, it was a bit surprising that no significant group difference was found in mMRC scores but there were significantly lower D-12-C scores in the experimental group (Table 2; Fig 2E, 2F). Lower D-12-C scores indicated less severe dyspnea during activities of daily living, suggesting that ACBT with WBBE could be particularly effective in alleviating the subjective burden of dyspnea. By encompassing both physical and affective domains of breathlessness, D-12-C could perhaps reflect the nuanced improvements brought about by the extra training with ACBT. Together with better QOL observed post-intervention (lower CAT scores), these findings suggested that the intervention could improve perceived well-being and daily function, underscoring the value of multidimensional assessments. The observed correlations among the outcome measures also highlighted the consistent, common trends that reflected improvements across measures (Table 3).

For clinical management of COPD, therapeutic approaches for mitigating the degree of pulmonary impairment, alleviating dyspnea symptoms, improving exercise capacity and endurance, and enhancing QOL are key aspects of non-pharmacologic rehabilitative interventions [4]. As a flexibly implemented approach, components of the ACBT cycle could be adjusted according to patient needs, in terms of the order, frequency and intensity of FET, thoracic expansion exercises, and relaxed breathing [8,9]. Together with thoracic expansion exercises and interspersed relaxed breathing, FET could facilitate the loosening and excretion of airway secretions with expiratory muscle training in coordination with glottal opening, leading to reduced airway obstruction and improvements in dyspnea [9,10]. To target our older adults (47 of 51 participants being older than 60), the specific ACBT cycle adopted consisted of 1–2 sets of FET and 3–4 sets of thoracic expansion exercises interspersed in between relaxed breathing, with relaxed breathing being emphasized given the likely need for increased rest for FET attempts in older individuals [8,11].

A key premise of this study was the combined effect of ACBT and WBBE (airway clearance plus exercises) being complementary to each other with exercises involving primarily respiratory muscles (ACBT) and respiratory plus non-respiratory muscles (WBBE), leading to improvements beyond what could be achieved alone. Our findings demonstrated that following 90 days of intervention, effect of the combined, extra training with ACBT was superior to that of WBBE alone, with significantly better pulmonary function, exercise capacity, dyspnea symptoms and QOL in the experimental group (Table 2; Fig 2). For future studies, it would be important to evaluate long-term effects of the combined training given the chronic nature of stable COPD, for instance with follow-up assessments 6 months and 12 months after completion of the intervention. It would also be interesting to evaluate if the combined training would be superior to the effect of ACBT alone as well.

Also, a study on the effect of respiratory muscle endurance training on athletes with high lesion level paraplegia revealed that endurance training could improve respiratory muscle endurance and ventilatory function without necessarily demonstrating changes in resting lung function (FEV1/FVC ratio), a pattern of findings that could be comparable to ours despite the very different study populations and different types of specific respiratory training implemented [33]. The comparable findings across the studies suggested that a common theme of intervention approaches focusing on respiratory muscle training could be applicable in the wider context of respiratory training, perhaps beyond COPD patients across discipline-specific training approaches.

### Limitations

One limitation of this study was the unbalanced duration of exercise training for the participant groups, with the experimental group undergoing two 30-minute sessions daily (on ACBT/WBBE), versus the control group undergoing only one 30-minute session daily (on WBBE). Yet, the unbalanced training duration (exercise dose) across groups might not have been a significant confounding factor, as the effect of duration of exercise training *alone* being not necessarily definitive on training outcomes given no significant differences were found between single and double daily training sessions [34], and between 8-week and 12-week intervention programs [35]. Paneroni et al. compared the effects of two endurance submaximal exercise protocols (one versus two daily 40-minute training sessions) in COPD patients, and found similar clinically meaningful improvements in dyspnea symptoms, 6MWT, and QOL [34]. Bishop et al. evaluated the effects of 8-week versus 12-week supervised PR program of endurance and strength training, and found equivalence in endurance exercise capacity (endurance shuttle walk test) across the programs [35]. In addition, a comprehensive review addressing the impact of exercise dose on cardiovascular health outcomes suggested that the effect of exercise training volume (exercise dose) *alone* remains inconclusive and a higher exercise dose itself may not lead to improved performance in a straightforward manner, at least not for the high end of the dose spectrum in terms of its effect on cardiovascular health [36]. Nevertheless, it would have been a cleaner experimental design with a balanced duration of exercise training across participant groups, and such design should be adopted in future studies. A second limitation was that participants in the experimental group had somewhat less severe COPD than those in the control group to begin with (mean GOLD stage of 1.48 versus 1.92), although this difference did not reach statistical significance. To achieve more unbiased comparisons, participants with more balanced GOLD staging across the two groups should be included for future studies. A third limitation was the difficulty of implementation of blinding for the participants, which could lead to performance bias in the participants especially for patient-reported outcome measures such as the D-12-C scores and CAT scores. Another limitation was that although significant group differences were found highlighting benefits of the combined training, the mechanism of such effect was not directly examined. It was not immediately clear whether the observed difference was in fact related to the premise that ACBT and WBBE would complement each other on respiratory versus non-respiratory muscles. For future studies, it would provide important insights by evaluating changes in activities of respiratory and non-respiratory muscles, through electromyographic recordings of respiratory muscle activities during ACBT, and limb muscle activities during WBBE. Finally, the effects of varying training frequency, intensity, and duration in ACBT implementation were not systematically investigated, which may be particularly relevant for optimizing outcomes in older adults with COPD. In future studies, one could explore what would be a proper frequency of relaxed breathing interspersed in between thoracic expansions and FET, in order to identify an optimized rest schedule for older adults.

## Conclusions

This study demonstrated that ACBT and WBBE could be effective complementary exercise training approaches for older adults with stable COPD, with extra training of ACBT as compared to WBBE alone resulting in significant improvements in pulmonary impairment, exercise capacity, dyspnea symptoms, and QOL, in particular for this specific combined protocol in an older Chinese cohort. However, the significance and implications of our findings are limited by the unbalanced exercise training dose across the participant groups, and experimental designs with more balanced exercise training should be adopted in future studies. Future studies should also explore how ACBT and WBBE could be better implemented through an optimal set of training frequency, intensity, and duration that could better fit the unique needs of older adults with stable COPD.

## Supporting information

S1 Data ACBT-COPD-paper-0PLOSOne-anonymized-raw-data.xlsx. (XLSX)

## References

[1] Agustí A, Celli BR, Criner GJ, et al. Global initiative for chronic obstructive lung disease 2023 report: GOLD executive summary. Eur Respir J. 2023;61(4):2300239. doi: 10.1183/13993003.00239-2023 36858443 PMC10066569

[2] Spruit MA, Singh SJ, Garvey C, ATS/ERS Task Force on Pulmonary Rehabilitation. An official American Thoracic Society/European Respiratory Society statement: key concepts and advances in pulmonary rehabilitation. Am J Respir Crit Care Med. 2013;188(8):e13–64. doi: 10.1164/rccm.201309-1634ST 24127811

[3] Troosters T, Janssens W, Demeyer H, Rabinovich RA. Pulmonary rehabilitation and physical interventions. Eur Respir Rev. 2023;32(168):220222. doi: 10.1183/16000617.0222-2022 37286219 PMC10245142

[4] Holland AE, Cox NS, Houchen-Wolloff L, et al. Defining modern pulmonary rehabilitation. An Official American Thoracic Society Workshop Report. Ann Am Thorac Soc. 2021;18(5): e12–e29. doi: 10.1513/AnnalsATS.202102-146ST PMC8086532 33929307

[5] O’Donnell DE, Milne KM, James MD, de Torres JP, Neder JA. Dyspnea in COPD: new mechanistic insights and management implications. Adv Ther. 2020;37(1):41–60. doi: 10.1007/s12325-019-01128-9 31673990 PMC6979461

[6] COPD Group of Chinese Thoracic Society, Chinese Medical Association, COPD Committee of Chinese Association of Chest Physician. Guidelines for the diagnosis and management of chronic obstructive pulmonary disease (revised version 2021). Chinese Journal of Tuberculosis and Respiratory Diseases. 2021;44(3):170–205. doi: 10.3760/cma.j.cn112147-20210109-00031 33721932

[7] Pryor JA, Webber BA, Hodson ME. Effect of chest physiotherapy on oxygen saturation in patients with cystic fibrosis. Thorax. 1990;45(1):77. doi: 10.1136/thx.45.1.77 2321184 PMC475668

[8] Pryor JA, Webber BA. Physiotherapy techniques. Physiotherapy for respiratory and cardiac problems. 2nd ed. Edinburgh: Churchill Livingstone; 1998. p. 137–55.

[9] Lewis LK, Williams MT, Olds TS. The active cycle of breathing technique: a systematic review and meta-analysis. Respir Med. 2012;106(2):155–72. doi: 10.1016/j.rmed.2011.10.014 22100537

[10] Wilson LM, Saldanha IJ, Robinson KA. Active cycle of breathing technique for cystic fibrosis. Cochrane Database Syst Rev. 2023;2(2):CD007862. doi: 10.1002/14651858.CD007862.pub5 36727723 PMC9893420

[11] Zisi D, Chryssanthopoulos C, Nanas S, Philippou A. The effectiveness of the active cycle of breathing technique in patients with chronic respiratory diseases: a systematic review. Heart Lung. 2022;53:89–98. doi: 10.1016/j.hrtlng.2022.02.006 35235877

[12] Celli BR, Cote CG, Marin JM, Casanova C, Montes de Oca M, Mendez RA, et al. The body-mass index, airflow obstruction, dyspnea, and exercise capacity index in chronic obstructive pulmonary disease. N Engl J Med. 2004;350(10):1005–12. doi: 10.1056/NEJMoa021322 14999112

[13] ATS Committee on Proficiency Standards for Clinical Pulmonary Function Laboratories. ATS statement: guidelines for the six-minute walk test. Am J Respir Crit Care Med. 2002;166(1):111–7. doi: 10.1164/ajrccm.166.1.at1102 12091180

[14] Mahler DA, Wells CK. Evaluation of clinical methods for rating dyspnea. Chest. 1988;93(3):580–6. doi: 10.1378/chest.93.3.580 3342669

[15] Yorke J, Moosavi SH, Shuldham C, Jones PW. Quantification of dyspnoea using descriptors: development and initial testing of the Dyspnoea-12. Thorax. 2010;65(1):21–6. doi: 10.1136/thx.2009.118521 19996336 PMC2795166

[16] Jones PW, Harding G, Berry P, Wiklund I, Chen W-H, Kline Leidy N. Development and first validation of the COPD assessment test. Eur Respir J. 2009;34(3):648–54. doi: 10.1183/09031936.00102509 19720809

[17] Boutron I, Altman DG, Moher D, Schulz KF, Ravaud P, CONSORT NPT Group. CONSORT statement for randomized trials of nonpharmacologic treatments: a 2017 update and a CONSORT extension for nonpharmacologic trial abstracts. Ann Intern Med. 2017;167(1):40–7. doi: 10.7326/M17-0046 28630973

[18] Graham BL, Steenbruggen I, Miller MR, et al. Standardization of spirometry 2019 update. An Official American Thoracic Society and European Respiratory Society Technical Statement. Am J Respir Crit Care Med. 2019;200(8):e70–88. doi: 10.1164/rccm.201908-1590ST PMC6794117 31613151

[19] Zhu L, Chen RC. Chinese experts’ consensus on the standardization of adult routine spirometry. Clin Pulm Med. 2022;27(11):1621–33. doi: 10.3969/j.issn.1009-6663.2022.11.001

[20] Puente-Maestu L, Palange P, Casaburi R, Laveneziana P, Maltais F, Neder JA, et al. Use of exercise testing in the evaluation of interventional efficacy: an official ERS statement. Eur Respir J. 2016;47(2):429–60. doi: 10.1183/13993003.00745-2015 26797036

[21] Mahler DA, Rosiello RA, Harver A, Lentine T, McGovern JF, Daubenspeck JA. Comparison of clinical dyspnea ratings and psychophysical measurements of respiratory sensation in obstructive airway disease. Am Rev Respir Dis. 1987;135(6):1229–33. doi: 10.1164/arrd.1987.135.6.1229 3592398

[22] Hajiro T, Nishimura K, Tsukino M, Ikeda A, Koyama H, Izumi T. Analysis of clinical methods used to evaluate dyspnea in patients with chronic obstructive pulmonary disease. Am J Respir Crit Care Med. 1998;158(4):1185–9. doi: 10.1164/ajrccm.158.4.9802091 9769280

[23] Zhang R, Tan X, He Q, Chen Q, Gai J, Wei J, et al. Comparison of symptom and risk assessment methods among patients with chronic obstructive pulmonary disease. Chin Med J (Engl). 2014;127(14):2594–8. 25043073

[24] Celli BR. Predictors of mortality in COPD. Respir Med. 2010;104(6):773–9. doi: 10.1016/j.rmed.2009.12.017 20417082

[25] Choi TCM, Chan LLY, Tsang HC, Vong YP, Cheng YK, To YL, et al. Adaptation and validation of the Chinese version of Dyspnoea-12 scale in individuals with chronic obstructive pulmonary disease. Clin Respir J. 2021;15(10):1081–7. doi: 10.1111/crj.13411 34145767 PMC8518647

[26] Chai J, Liu T, Cai B. Evaluation of clinical significance of chronic obstructive pulmonary disease assessment test. Zhonghua Jie He He Hu Xi Za Zhi. 2011;34(4):256–8. 21609607

[27] Analysis of Covariance (ANCOVA). Encyclopedia of Measurement and Statistics. Sage Publications, Inc.; 2007. doi: 10.4135/9781412952644.n18

[28] Seveso A, Campagner A, Ciucci D, Cabitza F. Ordinal labels in machine learning: a user-centered approach to improve data validity in medical settings. BMC Med Inform Decis Mak. 2020;20(Suppl 5):142. doi: 10.1186/s12911-020-01152-8 32819345 PMC7439656

[29] Agresti A. An Introduction to Categorical Data Analysis. 3rd ed. Wiley. 2018.

[30] Zhou M, Wang H, Zeng X, Yin P, Zhu J, Chen W, et al. Mortality, morbidity, and risk factors in China and its provinces, 1990-2017: a systematic analysis for the Global Burden of Disease Study 2017. Lancet. 2019;394(10204):1145–58. doi: 10.1016/S0140-6736(19)30427-1 31248666 PMC6891889

[31] Cannon MF, Goldfarb DG, Zeig-Owens RA, Hall CB, Choi J, Cohen HW, et al. Normal lung function and mortality in world trade center responders and national health and nutrition examination survey III participants. Am J Respir Crit Care Med. 2024;209(10):1229–37. doi: 10.1164/rccm.202309-1654OC 38163381 PMC12042200

[32] Puhan MA, Mador MJ, Held U, Goldstein R, Guyatt GH, Schünemann HJ. Interpretation of treatment changes in 6-minute walk distance in patients with COPD. Eur Respir J. 2008;32(3):637–43. doi: 10.1183/09031936.00140507 18550610

[33] Fischer G, Tarperi C, George K, Ardigò LP. An exploratory study of respiratory muscle endurance training in high lesion level paraplegic handbike athletes. Clin J Sport Med. 2014;24(1):69–75. doi: 10.1097/JSM.0000000000000003 24326928

[34] Paneroni M, Vogiatzis I, Belli S, Savio G, Visca D, Zampogna E, et al. Is two better than one? The impact of doubling training volume in severe COPD: a randomized controlled study. J Clin Med. 2019;8(7):1052. doi: 10.3390/jcm8071052 31323895 PMC6678655

[35] Bishop JA, Spencer LM, Dwyer TJ, McKeough ZJ, McAnulty A, Leung R, et al. Effect of pulmonary rehabilitation duration on exercise capacity and health-related quality of life in people with chronic obstructive pulmonary disease (PuRe Duration Trial): a randomized controlled equivalence trial. Respirology. 2025;30(1):41–50. doi: 10.1111/resp.14820 39228164 PMC11688624

[36] Wasfy MM, Baggish AL. Exercise dose in clinical practice. Circulation. 2016;133(23):2297–313. doi: 10.1161/CIRCULATIONAHA.116.018093 27267537 PMC4902280

